# Angiogenic cytokines and their influence on circulating tumour cells in sera of patients with the primary diagnosis of breast cancer before treatment

**DOI:** 10.1186/s12885-016-2612-7

**Published:** 2016-07-27

**Authors:** Theresa Vilsmaier, Brigitte Rack, Wolfgang Janni, Udo Jeschke, Tobias Weissenbacher, SUCCESS Study Group

**Affiliations:** 1 Department of Obstetrics and Gynecology, Ludwig-Maximilians-University of Munich, Maistrasse 11, 80337 Munich, Germany; 2 Department of Gynecology and Obstetrics, University Hospital Ulm, Ulm, Germany

**Keywords:** Breast cancer, Vascular markers, sFLT1, PlGF

## Abstract

**Background:**

Circulating tumour cells (CTCs) have been found to be a prognostic marker for reduced disease free survival, breast cancer–specific survival, and overall survival before the start of systemic treatment.

**Methods:**

A total of 200 patients’ sera were included in this study, 100 patients being CTC positive and 100 patients being CTC negative. Matching criteria were histo-pathological grading, lymph node metastasis, hormone receptor status, TNM classification and survived breast cancer patients vs. deceased tumor associated patients. A multi cytokine/chemokine array was used to screen the sera for the angiogenic markers.

**Results:**

Statistical significant correlation was exposed for sFlt1 values in regard to the CTC-Status. CTC negative patients displayed increased sFlt1 expression opposed to CTC positive breast cancer patients. Furthermore, significant enhanced PIGF values were also disclosed in CTC negative patients compared to patients being CTC positive. Analyzing the living patient collective we found significant differences in sFlt1 and PlGF values in regard to CTC negative and CTC positive patients.

**Conclusion:**

Both vascular markers showed enhanced expression in the CTC negative patient collective. To continue, the collective graded G2 showed significantly enhanced sFlt1 expressions amongst patients with no CTCs. Moreover, the patient collective with no lymph node metastasis and CTC negativity indicated statistically significant increased sFlt1 values. A functional interaction of sFlt1 and PlGF was found, suggesting that their overexpression in tumour cells inhibits CTCs entering the peripheral blood. Furthermore, in regard to CTC negativity, sFlt1 and PlGF values may potentially serve as predictive markers.

**Trial registration:**

The TRN of this study is NCT02181101 and the date of registration was the 4^th^ of June 2014. The study was retrospectively registered.

## Background

Worldwide, breast cancer is the most common tumour diagnosed in women with an estimated 1.7 million new breast cancer cases and 522,000 breast cancer deaths in 2012 [1]. Whereby, the survival of breast cancer patients is intensely associated with prognostic factors such as tumour size, hormone-receptor-profile and presence of metastases [2]. New approaches have also established a correlation between poor prognosis and the detection of Circulating tumour cells (CTCs) before the start of systemic treatment [3]. CTCs in the peripheral blood can be used as a prognostic marker for reduced disease free-, breast cancer specific-, and overall- survival before the start of systemic treatment [3–7]. The detection of CTCs shortly after commence of therapy even provide complementary information concerning treatment response [6]. The SUCCESS study was one of the first trials to indicate the strong prognostic importance, associated with a less favourable outcome, of CTCs in early breast cancer before commencing systemic adjuvant treatment and after adjuvant chemotherapy in a large patient cohort [8].

It is increasingly evident that not only the breast cancer cells itself, but also the microenvironment of the tumour plays a significant role in terms of tumour progression, metastasis formation and treatment response [9]. To continue, tumour angiogenesis acts as a crucial factor in the microenvironment in the development and progression of breast cancer. In correspondence to the arising significance of CTC involvement in cancer therapy, the aim of this study was the evaluation of tumour-angiogenesis markers in association to CTC involvement. Furthermore, vascular markers could act as indicators for the absence or presence of CTCs, as the determination of CTCs is a time intense and expensive technique.

Neo-angiogenesis, the process of new blood capillary formation from pre-existing vessels, acts as a fundamental part in both embryonic and postnatal development, in the remodelling of various organ structures, and in particular in tumour growth [10]. Its precarious involvement with tumour evolution and penetration has already become a promising focus in cancer therapy [2]. It is implied that angiogenesis in tumours is part of a multistep progression including the signalling between breast cancer cells and several cell types within the tumours microenvironment [2, 10]. A range of pro-angiogenic cytokines, which succumb an overexpression of factors by the tumour, induces Angiogenesis [11]. One of the best described is the vascular endothelial growth factor (VEGF). This process of neo-vascularisation is also referred to as the “angiogenic switch” [2, 12]. This describes the transition of tumour cells, where the balance between pro- and anti-angiogenic factors lean towards pro-angiogenic markers, designating a progression to an expanding vascularized tumour and eventually to malignant behaviour [11–13]. Consequently our intention was to analyse the distribution of angiogenic markers: sFlt1, PlGF, VEGF, VEGF-C and VEGF-D and disclose the differences of their expression in breast cancer patients of the SUCCESS study group in terms of CTC involvement, histo-pathological grading, lymph node metastasis, hormone receptor status, TNM classification and survived breast cancer patients vs. deceased tumour associated patients. A Sandwich immunoassay ELISA and anti-species Multi-Array 96 well plates were used to screen the blood serum samples that enabled us to screen for all mentioned vascular markers in just one well at the same time.

The cytokines belonging to the vascular endothelial growth factor (VEGF) family and its important involvement in angiogenesis have been subject of major interest. The VEGF family includes six related gene members; VEGF, VEGF-B, VEGF-C, VEGF-D, VEGF-F and placental growth factor (PIGF) that are regulators of angiogenesis or lymphangiogenesis or of both processes [2, 11, 14]. To continue, the markers have been described to bind with diverse affinity to one of the three tyrosine kinase receptors known as vascular endothelial growth factor receptor VEGFR-1 (sFlt1), VEGFR-2 and VEGFR-3 [15–17], initiating a signalling cascade promoting survival, growth and migration of tumour cells [2, 18]. Increased levels of VEGF in tumour patients have been described as a well-established indicator of poor prognosis [19]. PIGF and sFlt1 on the other hand have been known to play a major role in preeclampsia, and even associated with a lower breast cancer risk later in life of those patients [20, 21].

In conclusion, the assessment of vascular tumour angiogenesis markers in relationship to CTC involvement and the expression of angiogenesis markers in terms of histo-pathological grading, lymph node involvement, hormone receptor status, TNM classification and survived breast cancer patients vs. deceased tumour associated patients, could found an advantage in regard to assessing the discrete risk of patients at the time of primary diagnosis. The assessment of the angiogenesis factors in patients with different phenotype breast cancer, could furthermore allow a profounder understanding of how angiogenesis-related genes may influence breast carcinogenesis, thus allowing an increased enhanced individualized treatment.

## Methods

### Study design and ethical board permission

Eligible patients were defined as women with breast cancer (stages pT1–T4, pN0–N3, M0) who accepted to participate in the phase I SUCCESS study (www.success-studie.de). SUCCESS was a prospective, randomized adjuvant study comparing three cycles of fluorouracil-epirubicin-cyclo-phosphamide (FEC; 500/100/500 mg/m2) followed by 3 cycles of docetaxel (100 mg/m2) every 3 weeks vs. three cycles of FEC followed by 3 cycles of gemcitabine (1000 mg/m2 d1,8)-docetaxel (75 mg/m2) each 3 weeks. After the completion of chemotherapy, the patients were furthermore randomized to receive either 2 or 5 years of zoledronate. Hormone receptor–positive women moreover received suitable endocrine treatment. The research questions related to CTC analysis, the blood sampling time points, and the methodology were prospectively designed, and the prognostic value of the CTCs was described as a scientific objective of the study protocol. The study was permitted by 37 German ethical boards (lead ethical board: LMU, Munich) and conducted in agreement with the Declaration of Helsinki.

Blood samples for CTC enumeration were collected from 2090 consecutive patients after complete resection of the primary tumour and before adjuvant chemotherapy after written informed consent was acquired. Nevertheless, sixty-four patients were disqualified because of test failure or a time intermission of more than 96 h between the blood collection and sample preparation. A follow-up evaluation after chemotherapy and before the beginning of endocrine or bisphosphonate treatment was available for a subgroup of 1492 patients (see homepage: http://www.success-studie.de).

### Patients

In this study 200 Patients of the SUCCESS study were incorporated and assigned into two groups: 100 Patients were CTC positive (Group 1. CTC Positive) and the other 100 Patients were CTC negative (Group 2. CTC Negative). These two groups were then framed and investigated correspondingly. Patients from respectively groups were then matched into pairs of two rendering to histo-pathological grading, lymph node involvement, hormone receptor type, TNM classification and survived patients vs. deceased patients breast cancer associated. The 200 patient samples that were investigated contained 160 patients that were still alive at last observation after end of therapy and 40 patients that had deceased during therapy tumour associated. Furthermore, the groups considered contained 98 patients graded G2 and 102 patients graded G3. Matching criteria of the 200 breast cancer patients did not allow patients graded G1. Tumour stage of the anamnestic diagnosis was categorised according to the TNM-classification, which was conducted correspondingly to the WHO System [22]. The matching of patients was executed according to the criteria at the time of primary diagnosis. The histo-pathological grading was classified conferring to the Bloom and Richardson system classification [23].

### Collection of blood samples and Detection of CTCs

Method was conducted as defined by the SUCCESS Study group [8]. CTCs were examined using the CellSearch System (Veridex, Raritan, NJ). Peripheral blood was drawn into three CellSave tubes (3x10 mL – Serum Vacutainer from BD Ref. Nr. 367896), sent at room temperature to the central laboratory at the University of Munich, and inspected within 96 h of collection. Consequently, the patient sera was frozen at −80 °C and seasoned in Nitrogen for long-term storage.

The patient blood samples were then centrifuged for 10 min at 800 × *g*. The plasma was removed, and a dilution buffer was added. This arrangement was overlaid on 6 mL of Histopaque (Sigma, Steinheim, Germany) and centrifuged for 10 min at 400 × *g*. Subsequently, 7.5 mL of this sample enclosing the buffy coat was treated on the CellTracks AutoPrep system using the CellSearch Epithelial Cell Kit (Veridex). After immuno-magnetic enrichment with an anti-Epcam antibody, the cells were marked with fluorescent anticytokeratin (CK8, 18, 19–phycoerythrin) and anti-CD45 antibodies (CD45–allophycocyan), and 4,6-diamidino-2-phenylindoledihy-drochloride was used to classify the intact cells.

The identification, documentation and enumeration of CTCs were achieved using the CellTracks Analyzer II. CTCs were stated as nucleated cells lacking CD45 and expressing cytokeratin. Two independent investigators assessed all positive samples. The samples with a minimum of one CTC per 30 mL of blood were considered as CTC positive.

### Measurement of cytokines

ELISA was performed with recently developed multi cytokine/chemokine arrays (Meso Scale Discovery®, Rockville, USA) to screen the blood serum samples for the vascular markers sFlt1, PIGF, VEGF, VEGF-C and VEGF-D. The immunoassays were commercially available. We used anti-species MULTI-ARRAY 96-well plates for the development of a sandwich immunoassay. Each assay in the panel was verified individually for the Specificity by running single calibrator with single detection antibodies. Non-specific binding levels were less than 0.5 % for all assays. The 10 spot MULTI-SPOT plates were pre-coated with capture antibodies on independent and well defined spots that allowed us to immobilize a primary capture antibody against our protein of interest - specific for one of each vascular marker. Standards and samples were added to the appropriate wells. A standard curve was furthermore run with each assay. We firstly added the blood serum, calibrator and control. After that we incubated at room temperature with shaking for 2 h. After eliminating excess samples from the well with wash buffer, we added a solution containing the detection (anti-target) antibody conjugated with electrochemiluminescent labels over the course of two incubation periods. During incubation time, where time slots differed in each test, the target present in the sample bound to the capture antibody immobilized on the working electrode surface by the anti-species antibody. Recruitment of the labelled detection antibody by the bound target completed the sandwich. After a second shaking incubation period (time differed for each test) wash buffer was used to eliminate the entire unbound enzymes and a MSD Read Buffer was added to produce the suitable chemical environment for electrochemiluminescence. We then loaded the plate into an MSD instrument (MESO QuickPlex SQ 120) for examination where voltage applied to the plate electrodes caused the captured labels to emit light. The instrument calculated the intensity of the emitted light to present a quantitative measure of the amount of the protein of interest that was present in the sample [24, 25] (see homepage: www.mesoscale.com).

### Statistical analysis

Statistical analysis was implemented using SPSS 22.0 (SPSS Inc., IBM, Chicago, IL). The outcomes collected were recorded and inserted into the SPSS database in the implied manner. We evaluated the relationship between each vascular marker: sFlt1, PIGF, VEGF, VEGF-C and VEGF-D and each matching criteria (1. CTC-Positive vs. CTC-Negative, 2. Patient survived vs. Patient deceased, 3. Grading G2 vs. Grading G3, 4. Lymph node involvement vs. No lymph node involvement, 5. Triple positive vs. Triple negative 6. Progesterone receptor-positive vs. Progesterone receptor-negative, 7. Oestrogen receptor-positive vs. Oestrogen receptor-negative, 8. Her 2/neu receptor-positive vs. Her 2/neu receptor-negative) in the total patient collective and also regarding each matching criteria alone, by the use of the non-parametric Spearman correlation coefficient. Each parameter to be considered needed to have a p value <0.50. Statistical significant results within the Spearman correlation coefficient were then additionally assessed with the non-parametric Mann-Whitney-*U*-test. Moreover, variables were scrutinized by the use of Box-Plot analysis. All statistical tests were considered significant at *p* < 0.05.

## Results

### CTC positive vs. CTC negative

**Fig. 1:**
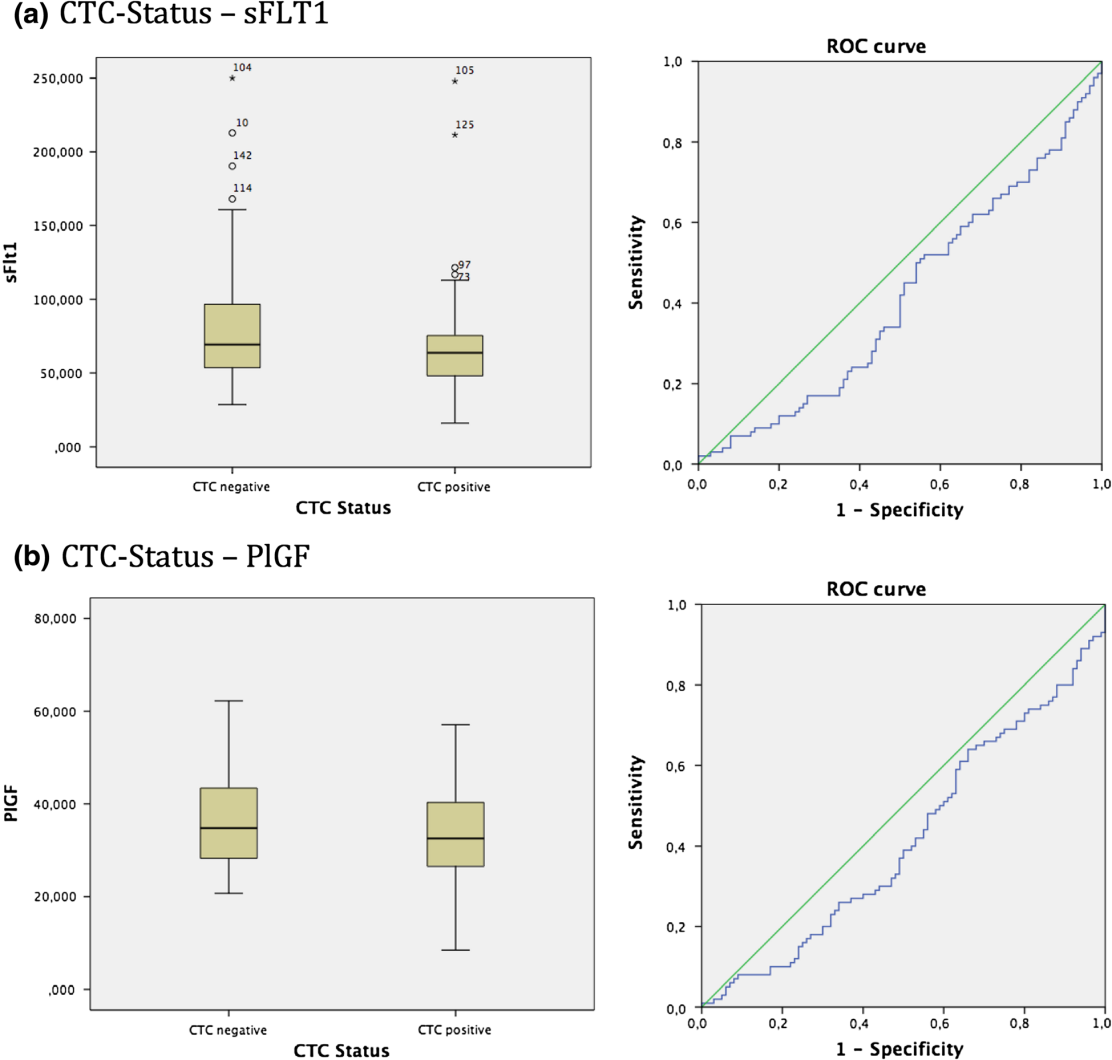
**a**: Box plot analysis of sFLT1 expression in sera of breast cancer patients. We identified a significant enhanced sFLT1 release in CTC negative patients compared to patients being CTC positive *p* = 0.034. In addition ROC analysis was performed. AUC value is 0.413. **b**: Box plot analysis of PlGF expression in sera of breast cancer patients. We identified a significant enhanced PlGF values in CTC negative patients compared to patients being CTC positive *p* = 0.043. In addition ROC analysis was performed. AUC value is 0.417

In the total patient collective, statistical significant differences were shown for sFlt1 values in regard to the CTC-Status. Box-plot analysis revealed that CTC negative patients exposed increased sFlt1 expression opposed to the CTC positive breast cancer patients that showed decreased sFlt1 values. The spearman correlation coefficient assessed the p-value of 0.034, additionally supported by the Mann-Whitney-*U*-Test *p* = 0.034, proving a significant correlation between CTC-status and sFlt1. In addition, ROC analysis was performed, exposing an AUC value of 0.413 (see Fig. 1a).

Furthermore, a statistical significant correlation was found for PIGF values concerning the CTC-status. Box-plot analysis identified significant enhanced PIGF values in CTC negative patients compared to patients being CTC positive. The spearman correlation coefficient assessed the p-value of 0.043 which was moreover supported by the Mann-Whitney-*U*-Test *p* = 0.043. ROC analysis implemented an AUC value of 0.417. (see Fig. 1b).

Nevertheless, the statistical analysis confirmed no significant correlation in terms of the CTC-Status regarding the vascular markers VEGF, VEGF-C, VEGF-D.

### Patient survived vs. patient deceased

**Fig. 2:**
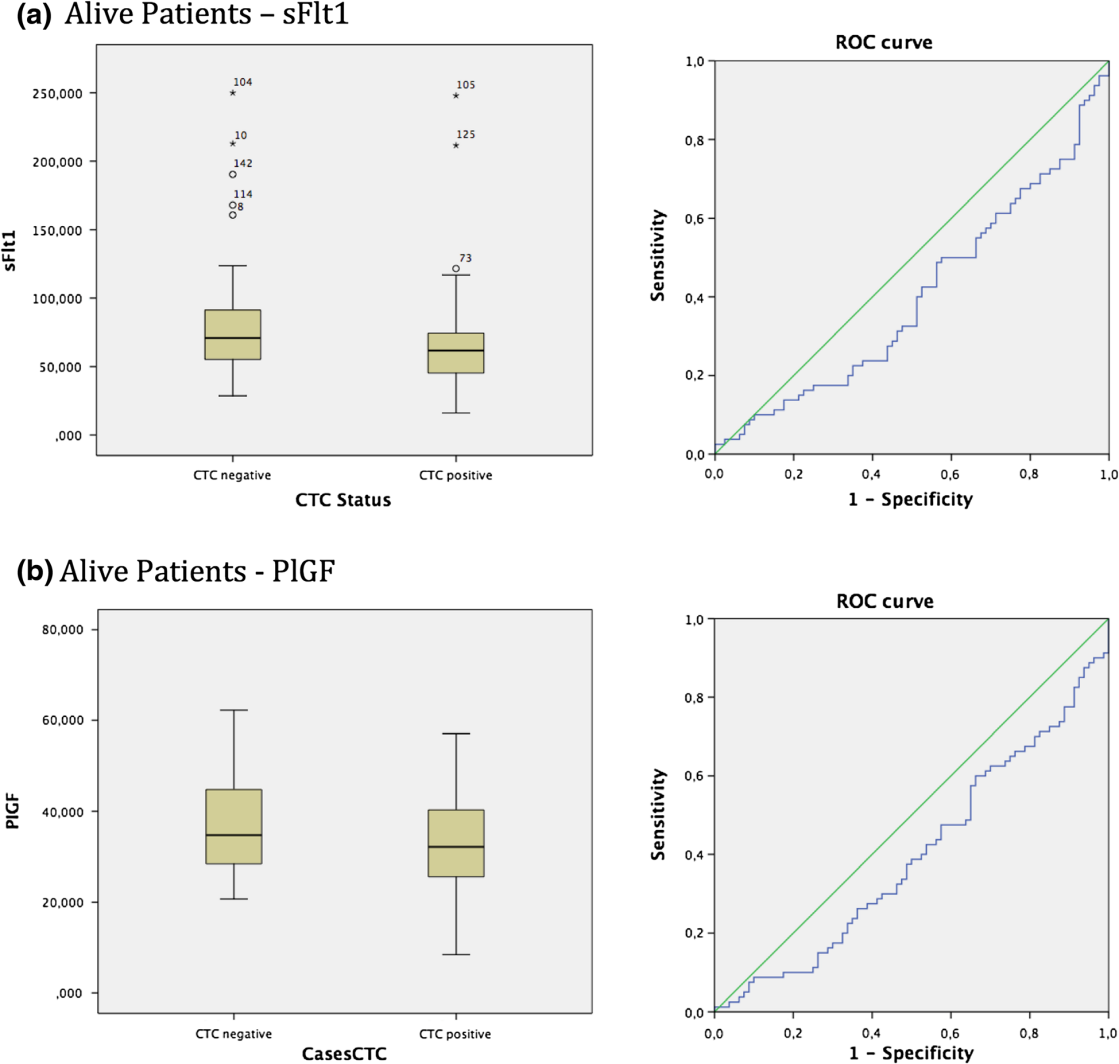
**a**: Box plot analysis of sFLT1 expression in sera of breast cancer patients who survived. We identified significant enhanced sFLT1 release in the survived patient collective and patients being CTC negative *p* = 0.030. In addition ROC analysis was performed. AUC value is 0.401. **b**: Box plot analysis of PlGF expression in sera of breast cancer patients who survived. We identified significant enhanced PlGF release in the survived patient collective and patients being CTC negative *p* = 0.026. In addition ROC analysis was performed. AUC value is 0.398

Analysing the patient collective who were still alive, and did not decease breast cancer associated, these showed statistically significant differences between CTC negative and CTC positive patients in terms of the vascular marker sFlt1. The box-plot analysis revealed that the survived patients collective who were CTC negative display higher levels of sFlt1 compared to the reduced values of sFlt1 in the survived patients with the presence of CTCs. The spearman correlation coefficient assessed the *p*-value of 0.030 which was additionally supported by the Mann-Whitney-*U*-Test *p* = 0.030. To continue, ROC analysis was performed, displaying an AUC value of 0.401 (see Fig. 2a).

Moreover, a statistical significant correlation was also proven for survived breast cancer patients being either CTC negative or CTC positive in respect to the vascular marker PIGF. The box-plot analysis disclosed that the survived patients collective who were CTC negative demonstrate increased PIGF expression in comparison to the decreased PIGF values in the survived patients being CTC positive. The spearman correlation coefficient evaluated the p-value of 0.025 which was furthermore reinforced by the Mann-Whitney-*U*-Test *p* = 0.026. ROC analysis was executed, revealing an AUC value of 0.398 (see Fig. 2b).

However, the statistical analysis verified no significant correlation in the survived patient collective regarding the vascular markers VEGF, VEGF-C, VEGF-D. To conclude, statistical analysis also demonstrated no significant differences concerning the deceased patients who had died breast cancer associated, with and without the presence of CTCs, in respect to the vascular markers sFlt1, PIGF, VEGF, VEGF-C and VEGF-D.

### Lymph node involvement vs. no lymph node involvement

**Fig. 3:**
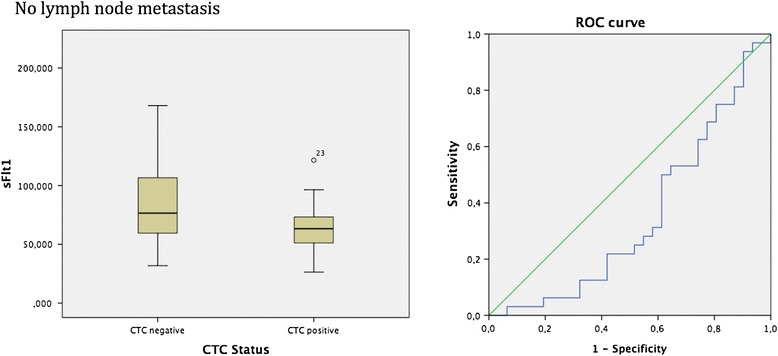
Box plot analysis of sFLT1 expression in sera of breast cancer patients with no lymph node metastasis. We identified significant enhanced sFLT1 release in the patient collective with no lymph node involvement and CTC negativity *p* = 0.041. In addition ROC analysis was performed. AUC value is 0.350

The patient collective with no lymph node metastasis indicated statistically significant differences between CTC negative and CTC positive breast cancer patients in regard to the vascular marker sFlt1. Box-plot analysis exposed that patients with no lymph node metastasis and CTC negativity demonstrated increased sFlt1 values in contrast to the reduced sFlt1 levels in patients with no lymph node metastasis and CTC positivity. The spearman correlation coefficient calculated the p-value of 0.039 which was furthermore sustained by the Mann-Whitney-*U*-Test *p* = 0.041. ROC analysis assessed the AUC value of 0.350 (see Fig. 3a).

Nonetheless, statistical analysis demonstrated no significant correlation concerning patients without lymph node involvement in respect to the vascular markers PIGF, VEGF, VEGF-C and VEGF-D. Furthermore, patients with lymph node metastasis, with and without the presence of CTCs, displayed no significant difference in respect to the vascular markers sFlt1, PIGF, VEGF, VEGF-C and VEGF-D.

### Grading G2 vs. Grading G3

**Fig. 4:**
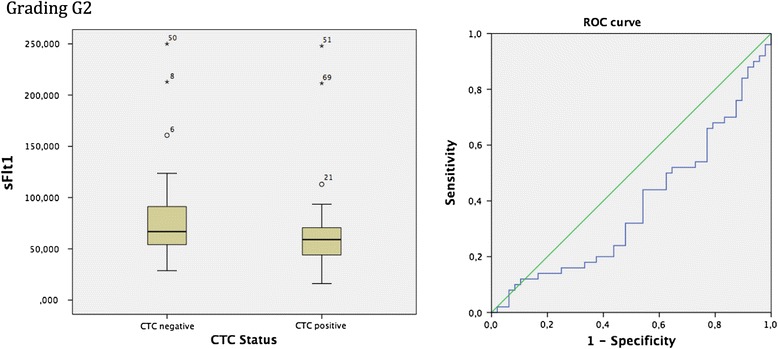
Box plot analysis of sFLT1 expression in sera of breast cancer patients with a G2 graded tumour. We identified significant enhanced sFLT1 release in the G2 graded patient collective and CTC negativity *p* = 0.042. In addition ROC analysis was performed. AUC value is 0.381

The collective graded G2 showed significant correlations amongst patients with the presence or absence of CTCs in terms of the vascular marker sFlt1. The box-plot analysis identified that patients graded with a G2 breast cancer and furthermore being negative for CTCs display higher levels of sFlt1 in comparison to the decreased values of sFlt1 in G2 graded breast cancer with the presence of CTCs. The spearman correlation coefficient evaluated the *p*-value of 0.041 which was additionally sustained by the Mann-Whitney-*U*-Test *p* = 0.042. To continue, ROC analysis was performed, revealing an AUC value of 0.381 (see Fig. 4a). None of the other vascular markers tested revealed differences in expression patterns in terms of Grading.

### Hormone receptor type presence vs. absence

Furthermore, the statistical analysis also verified no significant correlation regarding the presence or absence of each single hormone receptor type; Progesterone receptor, oestrogen receptor, Her2/neu, in patients with breast cancer with and without the presence of CTCs, with respect to the vascular markers sFlt1, PIGF, VEGF, VEGF-C and VEGF-D.

### Triple positive vs. triple negative

Moreover, no statistically significant correlation could be demonstrated with the general comparison of a triple negative hormone receptor to a triple positive breast cancer with and without the presence of CTCs in terms of the vascular markers sFlt1, PIGF, VEGF, VEGF-C and VEGF-D.

### Vascular marker correlation

**Fig. 5:**
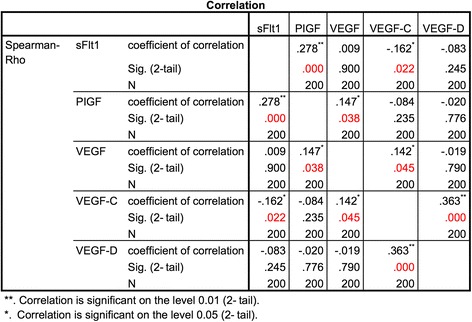
Spearman correlation of vascular markers: sFlt1, PIGF, VEGF, VEGF-C and VEGF-D. In regard to the total patient collective, significant correlation was found between: sFlt1 and PIGF (*p* = 0.000066), sFlt1 and VEGF-C (*p* = 0.022), PIGF and VEGF (*p* = 0.038), VEGF and VEGF-C (*p* = 0.045) and VEGF-C and VEGF-D (*p* = 0.0000001)

Significant correlation could be validated with the spearman correlation coefficient for the association between the vascular markers itself. The vascular markers examined were sFlt1, PIGF, VEGF, VEGF-C and VEGF-D. In regard to the total patient collective, with and without the presence of CTCs, significant correlations were found between sFlt1 and PIGF (*p* = 0.000066), sFlt1 and VEGF-C (*p* = 0.022), PIGF and VEGF (*p* = 0.038), VEGF and VEGF-C (*p* = 0.045), VEGF-C and VEGF-D (*p* = 0.0000001) (see Fig. 5).

## Discussion

Within this study we analysed the distribution of angiogenic markers: sFlt1, PIGF, VEGF, VEGF-C and VEGF-D and reveal the differences of their expression in the sera of breast cancer patients with and without circulating tumour cells. Significantly enhanced sFlt1 values were shown for the group of patients diagnosed with no CTCs. It is implied that sFlt1, which is the extra-cellular soluble domain of the VEGF receptor 1 (VEGFR1), traps VEGF thus acting as an important factor in the negative down regulation of angiogenesis [26, 27]. Furthermore, sFlt1 is described as an inhibiting factor of the pro-angiogenic VEGFR2 that acts as a positive signal conductor, whereas VEGFR-1 is a suppressor of VEGFR-2 signalling, consequently decreasing the neo-vascularisation stimulus [27, 28]. The tumour growth and the development of an invasive, aggressive and metastatic breast cancer are essentially reliant on the neo-vascularisation to provide blood supply for the nourishment and growth of the tumour. One can consequently suggest that the increased sFlt1 values in the CTC negative collective cause a significant disruption of tumour vascularisation, inhibiting CTCs being released into the peripheral blood. One could also suggest a significant additive inhibition delay in tumour growth, explaining and ensuing in the absence of CTCs in those patients.

This hypothesis is furthermore supported by increased levels of sFlt1 in the sera of the survived breast cancer patient collective and no CTC involvement. The ability of sFlt1 to bind and neutralize its target as it moves through the interstitial matrix is mostly described for one of the greatest powerful regulatory angiogenic agent VEGF [29, 30]. The role of sFlt1 has been previously shown to exercise a favourable outcome and advanced therapeutic effect in several tumour models [31]. Therefore, sFlt1 might have a major impact on anti-angiogenic activity by the requisitioning and neutralization of tumour secreted pro-angiogenic factors such as VEGF. This angiogenic inhibition might therefore contribute to slower and decreased tumour growth, inhibiting circulation specific metastasis, thus enhancing survival and resulting in a favourable conduct of those breast cancer patients.

To continue, the group of patients with no lymph node metastasis and no CTC involvement in the peripheral blood also indicated significantly enhanced sFlt1 expression. It is hypothesized that sFlt1 inhibits endothelial cell proliferation and sprouting in the tumours microenvironment, resulting in a decrease of the total number of tumour blood vessels, as well as the number of perfused vessels, demonstrating a combined anti-tumour and anti-vascular effect [9, 28]. The tumours microenvironment in association to sFlt1 values might therefore benefit from its anti-angiogenic effects by successfully inhibiting lymphangiogenesis. One can consequently suggest that sFlt1 not only acts as an important factor in terms of down regulating neo-vascularisation hence decreasing tumour progression, but also influences lymph metastasis formation in favour of patient outcome.

Furthermore, in the sera of breast cancer patients with a G2 histologically graded tumour and CTC negativity, showed significantly enhanced sFlt1 expression. It is implicated that sFlt1 leads to a significant delay in tumour growth without altering the revascularization of ischemic peripheral tissue [26, 32]. Also, sFlt1 presumably does not directly control growth of the malignant tumour cells but is linked with a more favourable outcome through the restriction of tumour vascularization [33]. Therefore, significantly enhanced sFlt1 expressions in CTC negative patients indicate a correlation with medium differentiated (G2) breast cancer patients and resulting in a favourable breast cancer manner.

Similarly to the sFlt1 values pattern, significantly enhanced Placental growth factor (PlGF) values were shown for the group of patients diagnosed with no CTCs in contrast to CTC positive breast cancer patients. Furthermore, increased levels of PlGF were also proven in the sera of the survived breast cancer patient collective and no CTC involvement. sFlt1 and PlGF values therefore correlate in these patient collectives, both showing enhanced values.

PlGF also belongs to the VEGF family, which exclusively binds to the sFlt1 receptor (VEGFR1) [34]. The association between PlGF and sFlt1 has already been established, especially in terms of preeclampsia. Placental production of sFlt1 is increased during preeclampsia [35, 36], whereas PlGF and VEGF are decreased during active disease and several weeks before commencement of symptoms [37]. sFlt1 hereby acts as a potent anti-angiogenic factor, binding and neutralizing the pro-angiogenic proteins VEGF and PlGF, playing a key role in the inhibition of placental angiogenesis [38]. Nevertheless, the role of PlGF in terms of tumour angiogenesis and tumour growth remains controversial. Some studies claim that PlGF promotes tumour angiogenesis and tumour growth [39–41], although numerous other analyses indicated that overexpression of PlGF in tumour cells suppresses tumour neovascularization and growth [42–47]. Research implies that in addition to forming homodimers, PlGF and VEGF can also form heterodimers that contrary to prior evidence may be inactive and function as inhibitors of tumour angiogenesis [11, 43, 48]. Therefore, PlGF may negatively modulate VEGF-induced angiogenesis by formation of biologically inactive heterodimers [43, 46, 49]. It is furthermore implied that PlGF in tumours significantly normalizes tumour vessels against vascular leakage, whereas blocking sFlt1 leads to an increased vascular leakage, thus causing a less favourable outcome [47]. Our results in terms of enhanced PlGF values in the CTC negative patient collective and furthermore in the survived breast cancer patient collective and no CTC involvement supports the implication that tumour derived PlGF negatively modulates tumour angiogenesis and tumour growth [47]. This hypothesis if furthermore supported by the increased levels of sFlt1 in these patient collectives as it is presumed that PlGF stimulates the proliferation of cell types that express sFlt1 [50]. Therefore one can suggest that the tumour derived PlGF in our breast cancer patients suppresses tumour angiogenesis, tumour growth and metastasis by a probable mechanism including PlGF homodimers or PlGF–VEGF heterodimers, activating a negative neovascularisation feedback via sFlt1 activation. Moreover, this hypothesis is reinforced by the significant correlations amongst the vascular markers. Significant correlation between sFlt1 and PlGF emphasize their association as well as the significant correlation between PlGF and VEGF, implying the presence of PlGF-VEGF heterodimers.

These findings demonstrate the functional interaction of sFlt1 and PlGF, suggesting that their overexpression in tumour cells inhibits CTCs entering the peripheral blood, thus emphasising a significant anti-angiogenic effect, inhibiting tumour growth and metastasis. Furthermore, in regard to CTC negativity, sFlt1 and PlGF values may potentially serve as predictive markers.

## Conclusion

Circulating tumour cells (CTCs) are a prognostic marker for reduced disease free survival, breast cancer–specific survival, and overall survival. CTC negative patients displayed increased sFlt1 expression opposed to CTC positive breast cancer patients. Furthermore, significant enhanced PIGF values were also disclosed in CTC negative patients compared to patients being CTC positive. In former studies, a functional interaction of sFlt1 and PlGF was found. Results of our study suggest that their overexpression in tumour cells inhibits CTCs entering the peripheral blood. Furthermore, in regard to CTC negativity, sFlt1 and PlGF values may potentially serve as predictive markers.

## Abbreviations

CTC, circulating tumour cells; sFlt1, soluble fms-like tyrosine kinase-1; PIGF, phosphatidylinositol-glycan biosynthesis class F protein; VEGF, vascular endothelial growth factor

## References

[1] Organization WH (2014). World Cancer Report 2014.

[2] Kristensen TB, Knutsson ML, Wehland M, Laursen BE, Grimm D, Warnke E, Magnusson NE (2014). Anti-Vascular Endothelial Growth Factor Therapy in Breast Cancer. Int J Mol Sci.

[3] Cristofanilli M, Budd GT, Ellis MJ, Stopeck A, Matera J, Miller MC, Reuben JM, Doyle GV, Allard WJ, Terstappen LW (2004). Circulating tumor cells, disease progression, and survival in metastatic breast cancer. N Engl J Med.

[4] Daskalaki A, Agelaki S, Perraki M, Apostolaki S, Xenidis N, Stathopoulos E, Kontopodis E, Hatzidaki D, Mavroudis D, Georgoulias V (2009). Detection of cytokeratin-19 mRNA-positive cells in the peripheral blood and bone marrow of patients with operable breast cancer. Br J Cancer.

[5] Bidard FC, Vincent-Salomon A, Sigal-Zafrani B, Dieras V, Mathiot C, Mignot L, Thiery JP, Sastre-Garau X, Pierga JY (2008). Prognosis of women with stage IV breast cancer depends on detection of circulating tumor cells rather than disseminated tumor cells. Ann Oncol.

[6] Hayes DF, Cristofanilli M, Budd GT, Ellis MJ, Stopeck A, Miller MC, Matera J, Allard WJ, Doyle GV, Terstappen LW (2006). Circulating tumor cells at each follow-up time point during therapy of metastatic breast cancer patients predict progression-free and overall survival. Clin Cancer Res.

[7] Botteri E, Sandri MT, Bagnardi V, Munzone E, Zorzino L, Rotmensz N, Casadio C, Cassatella MC, Esposito A, Curigliano G (2010). Modeling the relationship between circulating tumour cells number and prognosis of metastatic breast cancer. Breast Cancer Res Treat.

[8] Rack B, Schindlbeck C, Juckstock J, Andergassen U, Hepp P, Zwingers T, Friedl TW, Lorenz R, Tesch H, Fasching PA et al: Circulating tumor cells predict survival in early average-to-high risk breast cancer patients. J Natl Cancer Inst. 2014;106(5):pii: dju066. doi: 10.1093/jnci/dju066. 10.1093/jnci/dju066 PMC4112925 24832787

[9] Nienhuis HH, Gaykema SB, Timmer-Bosscha H, Jalving M, Brouwers AH, Lub-de Hooge MN, van der Vegt B, Overmoyer B, de Vries EG, Schroder CP. Targeting breast cancer through its microenvironment: Current status of preclinical and clinical research in finding relevant targets. Pharmacol Ther. 2015;147:63-79. doi: 10.1016/j.pharmthera.2014.11.004. Epub 2014 Nov 6. 10.1016/j.pharmthera.2014.11.004 25444756

[10] Harper J, Moses MA (2006). Molecular regulation of tumor angiogenesis: mechanisms and therapeutic implications. EXS.

[11] Neufeld G, Kessler O (2006). Pro-angiogenic cytokines and their role in tumor angiogenesis. Cancer Metastasis Rev.

[12] Baeriswyl V, Christofori G (2009). The angiogenic switch in carcinogenesis. Semin Cancer Biol.

[13] Hanahan D, Folkman J (1996). Patterns and emerging mechanisms of the angiogenic switch during tumorigenesis. Cell.

[14] Niu G, Chen X (2010). Vascular endothelial growth factor as an anti-angiogenic target for cancer therapy. Curr Drug Targets.

[15] Wehland M, Bauer J, Magnusson NE, Infanger M, Grimm D (2013). Biomarkers for anti-angiogenic therapy in cancer. Int J Mol Sci.

[16] Ferrara N (2005). The role of VEGF in the regulation of physiological and pathological angiogenesis. EXS.

[17] Ferrara N (2004). Vascular endothelial growth factor: basic science and clinical progress. Endocr Rev.

[18] Grimm D, Bauer J, Ulbrich C, Westphal K, Wehland M, Infanger M, Aleshcheva G, Pietsch J, Ghardi M, Beck M (2010). Different responsiveness of endothelial cells to vascular endothelial growth factor and basic fibroblast growth factor added to culture media under gravity and simulated microgravity. Tissue Eng A.

[19] Jelkmann W (2001). Pitfalls in the measurement of circulating vascular endothelial growth factor. Clin Chem.

[20] Vatten LJ, Romundstad PR, Jenum PA, Eskild A (2009). Angiogenic balance in pregnancy and subsequent breast cancer risk and survival: a population study. Cancer Epidemiol Biomarkers Prev.

[21] Gingery A, Bahe EL, Gilbert JS (2009). Placental ischemia and breast cancer risk after preeclampsia: tying the knot. Expert Rev Anticancer Ther.

[22] Fritz A, Percy C, Jack A, Shanmugaratnam K, Sobin L, Parkin DM, Whelan S (2000). International Classification of Diseases for Oncology 3.

[23] Elston EW, Ellis IO (1993). Method for grading breast cancer. J Clin Pathol.

[24] Mitsunaga S, Ikeda M, Shimizu S, Ohno I, Furuse J, Inagaki M, Higashi S, Kato H, Terao K, Ochiai A (2013). Serum levels of IL-6 and IL-1beta can predict the efficacy of gemcitabine in patients with advanced pancreatic cancer. Br J Cancer.

[25] Breitbart W, Rosenfeld B, Tobias K, Pessin H, Ku GY, Yuan J, Wolchok J (2014). Depression, cytokines, and pancreatic cancer. Psycho-Oncology.

[26] Verrax J, Defresne F, Lair F, Vandermeulen G, Rath G, Dessy C, Preat V, Feron O (2011). Delivery of soluble VEGF receptor 1 (sFlt1) by gene electrotransfer as a new antiangiogenic cancer therapy. Mol Pharm.

[27] Shibuya M (2013). Vascular endothelial growth factor and its receptor system: physiological functions in angiogenesis and pathological roles in various diseases. J Biochem.

[28] Bazan-Peregrino M, Sainson RC, Carlisle RC, Thoma C, Waters RA, Arvanitis C, Harris AL, Hernandez-Alcoceba R, Seymour LW (2013). Combining virotherapy and angiotherapy for the treatment of breast cancer. Cancer Gene Ther.

[29] Kim KJ, Li B, Winer J, Armanini M, Gillett N, Phillips HS, Ferrara N (1993). Inhibition of vascular endothelial growth factor-induced angiogenesis suppresses tumour growth in vivo. Nature.

[30] Rudge JS, Thurston G, Davis S, Papadopoulos N, Gale N, Wiegand SJ, Yancopoulos GD (2005). VEGF trap as a novel antiangiogenic treatment currently in clinical trials for cancer and eye diseases, and VelociGene- based discovery of the next generation of angiogenesis targets. Cold Spring Harb Symp Quant Biol.

[31] Kong HL, Hecht D, Song W, Kovesdi I, Hackett NR, Yayon A, Crystal RG (1998). Regional suppression of tumor growth by in vivo transfer of a cDNA encoding a secreted form of the extracellular domain of the flt-1 vascular endothelial growth factor receptor. Hum Gene Ther.

[32] Bodempudi V, Ohlfest JR, Terai K, Zamora EA, Vogel RI, Gupta K, Hebbel RP, Dudek AZ (2010). Blood outgrowth endothelial cell-based systemic delivery of antiangiogenic gene therapy for solid tumors. Cancer Gene Ther.

[33] Schmitz V, Kornek M, Hilbert T, Dzienisowicz C, Raskopf E, Rabe C, Sauerbruch T, Qian C, Caselmann WH (2005). Treatment of metastatic colorectal carcinomas by systemic inhibition of vascular endothelial growth factor signaling in mice. World J Gastroenterol.

[34] Dewerchin M, Carmeliet P (2014). Placental growth factor in cancer. Expert Opin Ther Targets.

[35] Maynard SE, Min JY, Merchan J, Lim KH, Li J, Mondal S, Libermann TA, Morgan JP, Sellke FW, Stillman IE (2003). Excess placental soluble fms-like tyrosine kinase 1 (sFlt1) may contribute to endothelial dysfunction, hypertension, and proteinuria in preeclampsia. J Clin Invest.

[36] Levine RJ, Maynard SE, Qian C, Lim KH, England LJ, Yu KF, Schisterman EF, Thadhani R, Sachs BP, Epstein FH (2004). Circulating angiogenic factors and the risk of preeclampsia. N Engl J Med.

[37] Tjoa ML, Levine RJ, Karumanchi SA (2007). Angiogenic factors and preeclampsia. Front Biosci.

[38] Remy S, Govarts E, Bruckers L, Paulussen M, Wens B, Hond ED, Nelen V, Baeyens W, van Larebeke N, Loots I (2014). Expression of the sFLT1 gene in cord blood cells is associated to maternal arsenic exposure and decreased birth weight. PLoS One.

[39] Odorisio T, Schietroma C, Zaccaria ML, Cianfarani F, Tiveron C, Tatangelo L, Failla CM, Zambruno G (2002). Mice overexpressing placenta growth factor exhibit increased vascularization and vessel permeability. J Cell Sci.

[40] Fischer C, Jonckx B, Mazzone M, Zacchigna S, Loges S, Pattarini L, Chorianopoulos E, Liesenborghs L, Koch M, De Mol M (2007). Anti-PlGF inhibits growth of VEGF(R)-inhibitor-resistant tumors without affecting healthy vessels. Cell.

[41] Van de Veire S, Stalmans I, Heindryckx F, Oura H, Tijeras-Raballand A, Schmidt T, Loges S, Albrecht I, Jonckx B, Vinckier S (2010). Further pharmacological and genetic evidence for the efficacy of PlGF inhibition in cancer and eye disease. Cell.

[42] Schomber T, Kopfstein L, Djonov V, Albrecht I, Baeriswyl V, Strittmatter K, Christofori G (2007). Placental growth factor-1 attenuates vascular endothelial growth factor-A-dependent tumor angiogenesis during beta cell carcinogenesis. Cancer Res.

[43] Eriksson A, Cao R, Pawliuk R, Berg SM, Tsang M, Zhou D, Fleet C, Tritsaris K, Dissing S, Leboulch P (2002). Placenta growth factor-1 antagonizes VEGF-induced angiogenesis and tumor growth by the formation of functionally inactive PlGF-1/VEGF heterodimers. Cancer Cell.

[44] Xu L, Cochran DM, Tong RT, Winkler F, Kashiwagi S, Jain RK, Fukumura D (2006). Placenta growth factor overexpression inhibits tumor growth, angiogenesis, and metastasis by depleting vascular endothelial growth factor homodimers in orthotopic mouse models. Cancer Res.

[45] Hedlund EM, Hosaka K, Zhong Z, Cao R, Cao Y (2009). Malignant cell-derived PlGF promotes normalization and remodeling of the tumor vasculature. Proc Natl Acad Sci U S A.

[46] Bjorndahl M, Cao R, Eriksson A, Cao Y (2004). Blockage of VEGF-induced angiogenesis by preventing VEGF secretion. Circ Res.

[47] Hedlund EM, Yang X, Zhang Y, Yang Y, Shibuya M, Zhong W, Sun B, Liu Y, Hosaka K, Cao Y (2013). Tumor cell-derived placental growth factor sensitizes antiangiogenic and antitumor effects of anti-VEGF drugs. Proc Natl Acad Sci U S A.

[48] Cao Y, Linden P, Shima D, Browne F, Folkman J (1996). In vivo angiogenic activity and hypoxia induction of heterodimers of placenta growth factor/vascular endothelial growth factor. J Clin Invest.

[49] Cao Y, Chen H, Zhou L, Chiang MK, Anand-Apte B, Weatherbee JA, Wang Y, Fang F, Flanagan JG, Tsang ML (1996). Heterodimers of placenta growth factor/vascular endothelial growth factor. Endothelial activity, tumor cell expression, and high affinity binding to Flk-1/KDR. J Biol Chem.

[50] Angelucci C, Lama G, Iacopino F, Maglione D, Sica G (2001). Effect of placenta growth factor-1 on proliferation and release of nitric oxide, cyclic AMP and cyclic GMP in human epithelial cells expressing the FLT-1 receptor. Growth Factors.

